# Mechanisms of Estrogens’ Dose-Dependent Neuroprotective and Neurodamaging Effects in Experimental Models of Cerebral Ischemia

**DOI:** 10.3390/ijms12031533

**Published:** 2011-02-25

**Authors:** Jakob O. Strom, Annette Theodorsson, Elvar Theodorsson

**Affiliations:** 1 Department of Clinical and Experimental Medicine/Clinical Chemistry, Linkoping University, SE-581 83 Linköping, Sweden; E-Mails: annette.theodorsson@liu.se (A.T.); elvar.theodorsson@liu.se (E.T.); 2 Department of Clinical and Experimental Medicine/Neurosurgery, Linkoping University, University Hospital, SE-581 85 Linkoping, Sweden

**Keywords:** estrogen, cerebral ischemia, stroke, animal experiments, administration methods

## Abstract

Ever since the hypothesis was put forward that estrogens could protect against cerebral ischemia, numerous studies have investigated the mechanisms of their effects. Despite initial studies showing ameliorating effects, later trials in both humans and animals have yielded contrasting results regarding the fundamental issue of whether estrogens are neuroprotective or neurodamaging. Therefore, investigations of the possible mechanisms of estrogen actions in brain ischemia have been difficult to assess. A recently published systematic review from our laboratory indicates that the dichotomy in experimental rat studies may be caused by the use of insufficiently validated estrogen administration methods resulting in serum hormone concentrations far from those intended, and that physiological estrogen concentrations are neuroprotective while supraphysiological concentrations augment the damage from cerebral ischemia. This evidence offers a new perspective on the mechanisms of estrogens’ actions in cerebral ischemia, and also has a direct bearing on the hormone replacement therapy debate. Estrogens affect their target organs by several different pathways and receptors, and the mechanisms proposed for their effects on stroke probably prevail in different concentration ranges. In the current article, previously suggested neuroprotective and neurodamaging mechanisms are reviewed in a hormone concentration perspective in an effort to provide a mechanistic framework for the dose-dependent paradoxical effects of estrogens in stroke. It is concluded that five protective mechanisms, namely decreased apoptosis, growth factor regulation, vascular modulation, indirect antioxidant properties and decreased inflammation, and the proposed damaging mechanism of increased inflammation, are currently supported by experiments performed in optimal biological settings.

## 1. Introduction

In the 1990s, several studies demonstrated neuroprotective effects of estrogens in animal models of cerebral ischemia [1–3]. This supported a hypothesis of estrogen neuroprotection that earlier had been postulated from the clinical observation that women are less likely to suffer from stroke compared to men, and that this protection diminishes by the advent of menopause [4]. Several previous epidemiological studies had also corroborated this hypothesis by indicating decreased stroke incidence in women on hormone replacement therapy [4]. Encouraged by the potential of estrogens as a mean of preventing illnesses including stroke and other cardiovascular diseases, substantial research efforts have been invested in further studies of the matter. However, later studies have been contradictory regarding estrogens’ effects on stroke, exemplified by the large randomized controlled trial Women’s Health Initiative, which was interrupted prematurely because of increased incidences of breast cancer, stroke and cardiovascular disease, thus apparently antagonizing the hypothesis that estrogens are neuroprotective (it requires mention that in the case of this study conjugated equine estrogens were used, and not 17β-estradiol that has been used in most animal trials) [5].

A few animal studies also reported increased ischemic damage from estrogens [6–11], in contrast to a large number of experiments in which neuroprotection was found [12–14]. This dichotomy concerning estrogens’ effects in animal models of cerebral ischemia was analyzed in a recent systematic review of rat studies, designating the dose and mode of estrogen administration as the culprits [15], which was later experimentally confirmed [16]. Slow-release pellets for subcutaneous implantation produced by the company IRA (all abbreviations are listed above) was identified as the only mode of estrogen administration which has led to increased ischemic lesions, plausibly due to the prolonged, supra-physiological plasma concentration peak (300–600 pg/mL, in comparison to the physiological 5–65 pg/mL) which characterizes these implants [17]. This is well in line with the concept of hormesis, stating that steroid hormones can have diametrically different actions in different concentration ranges [18]. The fact that estrogens seem to exert their effects via several different pathways, such as the classical nuclear receptors ER-α and ER-β, membrane-linked receptors and through direct molecular mechanisms further adds to the complexity, and could account for the hormesis phenomenon. The highest dosed pellets were in the abovementioned systematic review found to be most neurodamaging, while pellets containing lower doses of estrogens were more likely to be protective. The two other main methods of administering estrogens to rats; subcutaneous silastic capsules (generated serum concentrations of 40 pg/mL diminishing to 5 pg/mL in 42 days [17]) and subcutaneous injections (generated baseline 17β-estradiol concentrations of 10–110 pg/mL [17]), showed consistent neuroprotection in the doses tested (Table 1) [15].

Concerning mechanisms of estrogens’ effects in cerebral ischemia, there have been numerous explanations, mainly focusing on estrogens’ protective properties, but also with some suggestions of detrimental pathways. In the following sections, we will review the five most extensively investigated potential neuroprotective mechanisms, namely decreased oxidative stress (Section 2.1) decreased inflammation (2.2), decreased apoptosis (2.4), growth factor regulation (2.5) and vascular modulation (2.6), and the three suggested neurodamaging mechanisms increased oxidative stress (2.1), increased inflammation (2.2) and increased excitotoxicity (2.3). Each section consists of a brief summary of data supporting the mechanism hypothesis, when required also with a complementary description of pathways. Subsequently the estrogen administration methods used and in the cited studies are described, and it is discussed how well this matches with the notion that estrogens are protective in physiological doses and damaging in very high doses. Concerning the actual serum estrogen concentrations in the cited studies, it is unfortunately very uncommon that researchers do measurements of 17β-estradiol on more than one occasion during an experiment. Concentrations measured in a single blood sample provide very little information about the serum concentrations at the other time-points of the study. Therefore, presenting the (in the *vast* majority of cases) single measurements from the studies cited run the risk of misleading the reader and is therefore not done here. Further, analysis of minute amounts of 17β-estradiol, most often performed with radioimmunoassay, should—because of the difficulties in calibrating the methods and the large inter-assay variations—always be performed including serum from native, cycling female rats to obtain reference intervals, which sadly is even rarer [32]. However, it is important to bear in mind that even if blood levels are monitored, these only represent a crude estimate of the concentrations in the brain, where the actual effects take place.

*In vitro* experiments are cited throughout the review, although it should be noted that the concentrations of estrogens used are generally several orders of magnitude higher than in whole-animal experiments and therefore hard to interpret to *in vivo* conditions. Interestingly, the dose-dependent dichotomy of studies reporting protective *versus* damaging results found in whole-animal experiments is not found in cell culture experiments.

**Figure 1.**
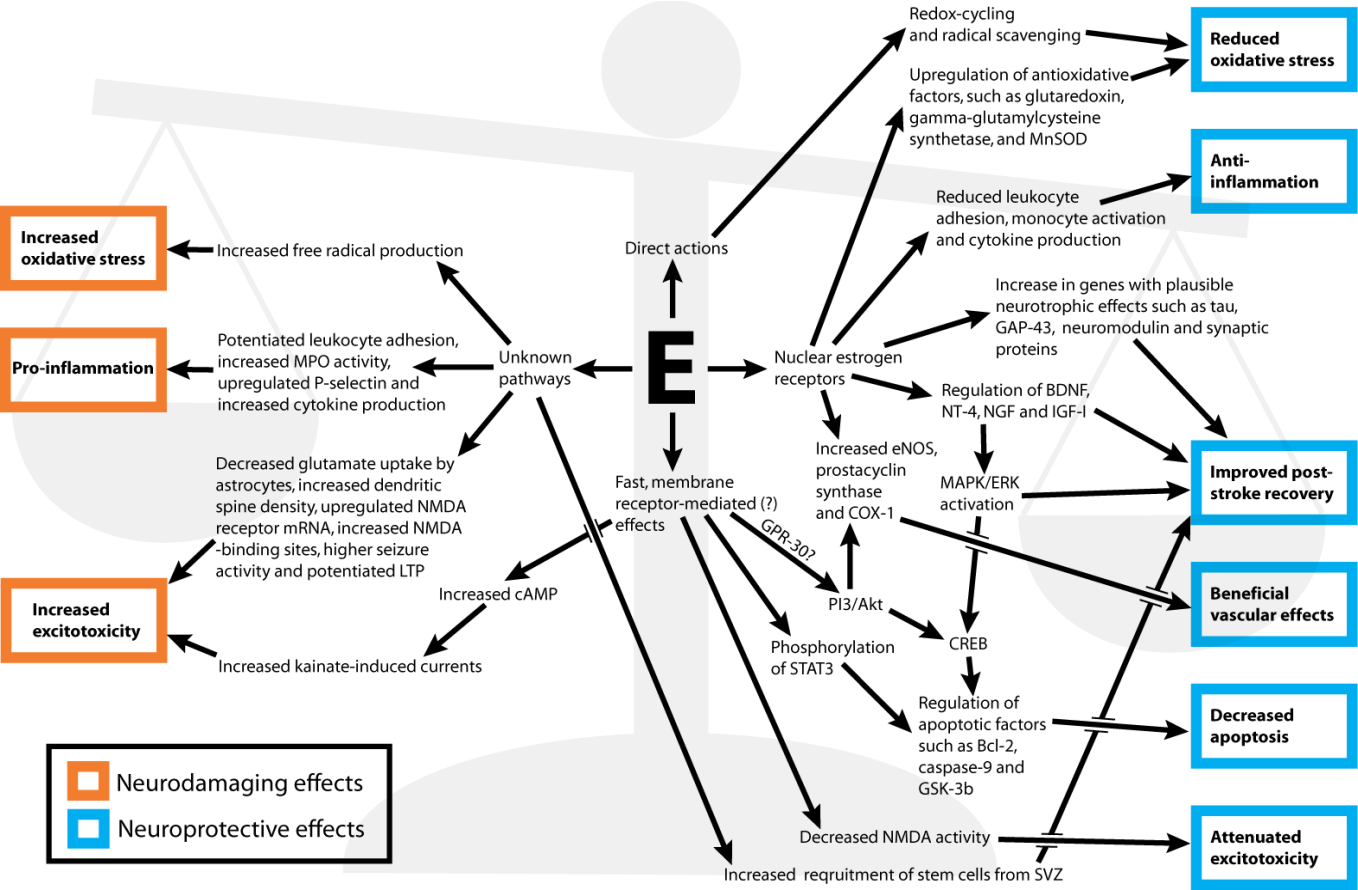
A simplified map of suggested pathways and mechanisms for estrogens’ effects in stroke. Orange and blue rectangles mark plausibly detrimental and protective effects, respectively. The balance in the background symbolizes that depending on the circumstances, such as the dose of estrogen, either the protective or detrimental mechanisms may dominate. The “E” in the middle of the figure is short for “Estrogens” (other abbreviations are detailed above the Introduction). Depicted pathways and mechanisms have previously been reviewed in numerous publications [12,13,34,37–43]. Each part of the figure is matched with exact citations in respective sections throughout the article.

Although not reviewed below, a number of additional suggested protective mechanisms also deserve mention, even though research efforts into their pathways are still in early stages. These include increased recruitment of stem cells from the subventricular zone [33], avoidance of apoptosis by balancing phosphatase activity [34] and decrease of excitotoxicity by reducing NMDA-signaling (please note that the opposite; that estrogens may increase excitotoxicity and thereby increase ischemic damage, is reviewed under 2.3) [35,36]. A simplified map of pathways and actions of estrogens that have been postulated to influence cerebral ischemia in a protective or detrimental direction is presented in Figure 1.

## 2. Mechanisms for Estrogens’ Neuroprotective and Neurodamaging Effects

### 2.1. Decreased and Increased Oxidative Stress as Mechanisms of Estrogen Neuroprotection and Neurodamage

Oxidative stress is an important mechanism in cellular damage in general and cerebral ischemia in particular. Ischemia prompts mitochondria to produce ROS, which causes direct damaging oxidative reactions such as lipid peroxidations, as well as triggering apoptotic cascades. The cell has intricate defense systems against oxidative damage, including scavenging activity by SOD, glutathione peroxidase, and catalase, and further detoxification by small molecules such as glutathione, ascorbic acid, and α-tocopherol. However, during cerebral ischemia, especially reperfusion, these systems are generally overrun by the massive oxidative stress [44]. Estrogens have been stipulated to exert their neuroprotective effects both through direct chemical effects and indirectly via upregulation of the cell’s anti-oxidative defense mechanisms (Figure 1) [34].

#### 2.1.1. Direct Anti-Oxidative Effects

Direct anti-oxidative effects have been found in several studies. More specifically, estrogens have been reported to prevent intracellular peroxide accumulation in an ER-independent manner [45], decrease ROS production [46], limit lipid peroxidation [47–50], protect against oxidative stress FeSO_4_ [51], and to decrease hydrogen peroxide concentrations [30]. In one of these studies, no extra protection was afforded by adding known potent free radical scavengers, indicating that estrogens exert all the protective effects available through anti-oxidative mechanisms [48]. Further, 17α-estradiol, a less feminizing enantiomer of 17β-estradiol, has been shown to protect against glutamate and hydrogen peroxide stress to a similar extent as 17β-estradiol, indicating the importance of receptor-independent pathways [52]. Anti-oxidative mechanisms have also been suggested merely on the basis that estrogens can protect against oxidative stress, although it should be emphasized that protection against an oxidative assault is not necessarily dependent on a primary anti-oxidative mechanism [53,54]. A further mechanism for estrogens’ direct anti-oxidative effect was proposed by Prokai *et al.*, providing evidence that estrogens can engage in a redox cycle in which estrogens turn into a quinol when eliminating a radical, to subsequently be converted back to the parent estrogen using NADPH as a reducing agent [55,56]. Interestingly, it was demonstrated that the quinol-cycling actually made the compound function as a prodrug that was selectively activated in the brain, importantly *without* uterotropic actions [55]. 17β-estradiol’s anti-oxidative effect has also been attributed to the hydroxyl group in the C3 position of the A ring, even though one study found no protection by neither of 2-hydroxyestradiol nor 2-methoxyestradiol, despite the fact that these estrogen metabolites have intact hydroxyl groups [57]. However, the last-mentioned study is difficult to assess because of possible differences in brain-uptake by the three compared compounds, and further, in another article it is on the contrary reported that 2-methoxyestradiol protects against ischemia [58].

Taken together, the current evidence indicates that estrogens exert direct anti-oxidative effects under certain circumstances. However, it requires emphasis that the genomic and non-genomic actions of estrogens are impossible to firmly separate, especially *in vivo*. Also, an important concern is that the abovementioned studies mainly have been performed *in vitro* and using hormone concentrations that are extremely high (in the magnitude of 0.1–100 μM) compared to what is normally achieved in animal models of cerebral ischemia [15,45,46,49,50]. An exception, which seems to support the anti-oxidative evidence, is a study by Kii *et al.*, in which estrogens were shown to decrease levels of hydrogen peroxide measured by microdialysis, which were not decreased by tamoxifen in a rat model of cerebral ischemia [30]. However, the results of this study remain to be confirmed in studies less fraught with assessment difficulties. Since estrogens are protective in cerebral ischemia only within a relatively narrow and low range of concentrations, studies showing the part played by estrogens anti-oxidative effects in physiological concentrations *in vivo* remain to be done. Thus, although estrogens are likely neurodamaging in high concentrations, even much higher levels, only relevant in cell cultures, seem to be required to produce the direct anti-oxidative effects of estrogens.

#### 2.1.2. Indirect Anti-Oxidative Effects

Indirect anti-oxidative effects of estrogens have been reported including attenuation of microglial superoxide release [59], increase of glutathione reductase, gamma-glutamylcystein synthetase, glutaredoxin and glutathione [60–64], increased MnSOD activity [65,66] and expression [67,68], upregulation of Cu/Zn SOD expression [67], reduction of free radical production via an increase mitochondrial efficiency [69,70], attenuation of NADPH oxidase activation [71,72] and decrease of the oxidative stress marker nitrotyrosine [67]. These effects have been found to at least in part result from nuclear ER-mediated upregulation of anti-oxidative proteins [34].

In contrast to the direct anti-oxidative mechanisms presented in the above section, these examples of upregulation of the oxidative defense system has been demonstrated in many studies in relevant biological contexts, such as mice and rats receiving subcutaneous and intraperitoneal injections [64,67,69–71]. Thus, at present the proposed indirect anti-oxidative mechanisms seem more likely to be relevant in actual whole-animal cerebral ischemia models than the direct anti-oxidative mechanisms do.

#### 2.1.3. Pro-Oxidative Effects

As aforementioned, estrogens have also been shown to *increase* oxidative stress, and thereby possibly augment ischemic damage (Figure 1) [9,73]. The reported pro-oxidative effects include increased mitochondrial ROS production [74,75], oxidative DNA-damage in sperm and ovarian surface epithelium [76,77], reduced levels of anti-oxidant proteins in rat brain [78], promotion of oxidative damage in rat liver cells [79] and increased ROS-production from the estrogen metabolites 2-methoxyestradiol and 4-hydroxyestradiol [80–82]. However, these pro-oxidative effects of estrogens have mainly been reported from *in vitro* experiments and in other tissues than the brain, while studies on the nervous system almost uniformly have found estrogens to exert anti-oxidant properties [73]. This could possibly reflect tissue-specific estrogen response patterns, which has been proposed to result from differences in cellular balance of ER-β *versus* ER-α [73].

Apart from a study by Pajovic *et al.* [78], studies demonstrating pro-oxidative properties of estrogens in a biological context relevant to cerebral ischemia are lacking. In this study, levels of glutathione peroxidase, glutathione-S-transferase and glutathione reductase in male rat brains were decreased in response to moderately dosed exogenous estrogen. Theoretically, this decrease is likely to hamper the cells’ anti-oxidative defense, increasing the risk of ischemia-induced cellular damage [78]. Even taking this study into account, the evidence for estrogenic pro-oxidative actions as a mechanism for increased damage in cerebral ischemia appears scarce, and it cannot be included as a plausible pathway for estrogens’ damaging effects.

### 2.2. Anti- and Pro-Inflammatory Actions as Mechanisms of Estrogen Neuroprotection and Neurodamage

Cerebral ischemia triggers an acute and prolonged inflammatory process in the brain, characterized by activation of microglia, production of inflammatory cytokines and infiltration of various inflammatory cells, including neutrophils, T-cells and monocytes/macrophages, into the damaged tissue. The inflammatory process is considered an important component of the pathophysiology of stroke, and especially the early inflammatory cell infiltration and cytokine production seem to be predominantly deleterious [83]. Experiments in rats have shown that intraventricular administration of TNF-α, IL-1 and IL-6 exacerbates stroke damage, suggesting a detrimental role of inflammation in the ischemic process [84–86]. Further support for this hypothesis is found in the observation that blockage of pro-inflammatory cytokines ameliorates ischemic damage [86–91].

#### 2.2.1. Anti-Inflammatory Effects

Anti-inflammatory properties of estrogens have been demonstrated in a large number of studies, and are commonly taken as important mechanisms for estrogens’ neuroprotective effects in stroke [86]. Estrogens have been shown to induce a wide range of anti-inflammatory effects via, for example, reducing leukocyte adhesion [92–94], decreasing pro-inflammatory cytokine production [95–102], decreasing monocyte activation [103] and altering the microglial activation pattern [104]. Both leukocytes and microglia express ER, offering a direct pathway for estrogens’ actions in inflammatory processes [86], and ER activation is e.g., thought to regulate iNOS transcription [105]. The classical pro-inflammatory cytokines IL-1, IL-6 and TNF-α lack ERE, but are thought to be affected by for example activated ER’s down regulation of nuclear c-Jun and JunD, leading to decreased occupation of AP-1 which in turn could increase the expression of TNF-α (Figure 1) [106].

The studies designed to investigate estrogens’ actions in inflammation have to a large extent been performed in cell cultures, where hormone concentrations are hard to extrapolate to concentrations in intact organisms. Of the studies performed in animals, most have focused on other organs than the brain, which potentially could lead to misinterpretation if the data are extrapolated to estrogens effects in cerebral ischemia. The effects of estrogens on inflammation are in many aspects organ specific, vividly exemplified by the estrogen-induced prostatitis in rats [107] in contrast to the amelioration of soft tissue inflammatory conditions [108]. To elucidate at which estrogen concentrations anti-inflammatory effects in the brain occur, we here narrow our focus to studies performed to assess effect on cerebral inflammation in animals. These are comparatively few, but include experiments that have shown that estrogens limit the activity of the pro-inflammatory transcription factor NFKB in a rat MCAo model [109], decrease leukocyte adhesion both before and after transient forebrain ischemia in rats [92], reduce number of microglia and astrocytes in mice [110], decrease cytokine production in animal models of MCAo [96] and NMDA-induced toxicity [97], block COX-2 activity and PGE2 production after IL-1β administration in rats [102], reduce iNOS activity [105], and decrease monocyte activation and recruitment in response to LPS [103]. In two studies, the importance of anti-inflammation for estrogens’ actions have been demonstrated by the lack of 17β-estradiol neuroprotection in iNOS knockout mice [111] and mice treated with the iNOS inhibitor aminoguanidine [112].

Of these studies, all but two have adopted presumptive low-dose or short-term 17β-estradiol regimes, such as various intraperitoneal or subcutaneous injection schedules and low-dose silastic capsules, which are in the dose range likely to induce protection against ischemic damage [15,92,95,96,102,103,105,109–112]. The remaining two of the abovementioned studies used pellets from IRA, which are high-dose regimes capable of inducing either protection or damage in cerebral ischemia [97,110]. In one of the studies using pellets [110], 17β-estradiol merely decreased the number of astrocytes and microglia without relation to stroke, which could be interpreted as a degenerative as well as an anti-inflammatory effect. Further, in the other high-dose pellet study, older rats given the same treatment showed *increased* cerebral inflammation [97]. Thus, of the studies reporting estrogen-induced decreases in animal brain inflammation, a majority have been performed with short-term or low-dose estrogens similar to regimens that previously have been reported to decrease cerebral ischemic damage, which is as expected if anti-inflammation is one of the actual protective mechanisms [15].

#### 2.2.2. Pro-Inflammatory Effects

Paradoxically, one of the suggested mechanisms for estrogens’ ability to *increase* damage in cerebral ischemia is the hormones’ *pro*-inflammatory capacity [7,37]. In several rat experiments, estrogens have been reported to potentiate leukocyte adhesion, increase P-selectin and MPO enzyme activity in cerebral ischemia [7,113], increase TNF-α, TLR-2 and IL-12 in response to LPS stress [114,115], increase IL-1β in a NMDA-toxicity model [97] and to worsen functional outcome in a model of chronic cerebral inflammation (Figure 1) [116].

Most interestingly, in sharp contrast to the majority of studies reporting decreased inflammation, all but one [113] of these studies adopted administration regimens that are likely to produce highly supraphysiological 17β-estradiol concentrations in the range that have been shown to exacerbate ischemic damage in rats [15,17]. The high-dose regimens used were slow-release capsules from IRA [7,97,115] and silastic capsules containing dissolved 17β-estradiol in concentrations about 10 [114] to 250 [116] times higher than the highest dissolved silastic capsule 17β-estradiol concentration that, to the best of our knowledge, has been reported to be neuroprotective [117]. The pro-inflammatory effects of estrogens have generally not been interpreted as resulting from the high hormone dose, but rather as synergistic effects of diabetes [7,113] and old age [97,115,118]. However, a possible contribution of factors such as age and disease do not explain the striking dominance of high-dose regimens in these experiments, thereby suggesting that estrogens in supraphysiological concentrations are likely to have a higher propensity for increasing inflammation, supporting the hypothesis that high-dose estrogens increase damage from cerebral ischemia. It should also be mentioned that estrogens indeed have been reported to protect both diabetic and old animals in several studies, contradicting a clear relation between age, diabetes and neurodamaging effects of estrogens [119–123].

### 2.3. Increased Excitotoxicity as a Mechanism of Estrogen Neurodamage

Excitotoxicity is a well-established feature in cerebral ischemia, and contributes to the pathophysiology by a series of events characterized by abnormal excitation by neurons due to pathological release of excitatory neurotransmitters from damaged cells. In the process, both NMDA and AMPA glutamate receptors are over stimulated, contributing to uptake of Na^+^, Cl^−^ and Ca^2+^ ions, which depolarizes neurons and leads to subsequent transmitter release, further stimulating receptors in a vicious cycle. The ion uptake leads to cellular edema and to activation of various detrimental Ca^2+^-dependent enzymes, which in turn damage the cell by degrading cytoskeletal proteins, damaging DNA and by increasing the generation of free radicals [124].

It has been stipulated that estrogens could augment the pathological process in cerebral ischemia by potentiating the excitotoxicity since estrogens have been reported to increase NMDA mRNA in the hippocampal CA1-region [125], increase NMDA-binding sites in CA1 [126,127], increase dendritic spine density or decreased ovariectomy-induced dendritic spine loss in CA1 [126,128–130], increased sensitivity of CA1 pyramidal cells to NMDA receptor-mediated synaptic input [126], facilitate seizure activity [131], augment LTP [132,133], increase the excitability of different neurons [134,135], decrease glutamate-uptake by astrocytes [136] and to facilitate kainite induced currents via cAMP-dependant phosphorylation (Figure 1) [137]. It is likely that a substance that facilitates NMDA activity and increases excitability could potentiate excitotoxicity and augment ischemic damage. In line with this hypothesis, it has been reported in several articles that decreased excitotoxicity, either by reducing the number of collaterals [138–140] or potentiating GABA-ergic transmission [141,142], is associated with amelioration of ischemic damage.

However, of the many aforementioned studies performed on animals, the vast majority have seen the potentially excitatory effects from low-dose or short-time estrogen administration regimens that are likely to protect from rather than increase ischemic damage [15,17,126,127,129,132,135]. None of the studies adopted the high-dose pellets from IRA that have been shown to be detrimental in cerebral ischemia. Thus it is as yet not established whether estrogens are able to increase excitotoxicity in doses that are relevant to the animal models that have reported *increased* ischemic damage from estrogens. Also, the abovementioned studies have merely presented indirect evidence of increased excitotoxicity by estrogens. In contrast, several studies have shown *decreased* excitotoxicity from estrogens in the same dose range (Figure 1) [36,143–149]. The studies indicating estrogen-induced increased excitotoxicity have notably largely been restricted to hippocampus, while the ischemic damage in the most common animal stroke model (MCAo) primarily involve the striatum and cerebral cortex.

In conclusion, the hypothesis that estrogens exacerbate ischemic damage by potentiating excitotoxicity has limited support since (1) the potentially excitotoxicity-increasing effects have mainly been demonstrated in experimental paradigms involving presumably neuroprotective hormone regimens; (2) no direct evidence of increased excitotoxicity is as yet available; (3) several studies have reported direct signs of *decreased* excitotoxicity from estrogen treatment; and (4) the studies reporting excitatory effects have largely been restricted to hippocampus, possibly reflecting site-specific effects.

### 2.4. Decreased Apoptosis as a Mechanism of Estrogen Neuroprotection

Apoptosis is a major pathophysiological mode of cell death in ischemic brain injury [150,151]. Ischemia triggers mitochondria to produce reactive oxygen species, which do not only directly damage lipids, proteins and nucleic acids in the cell, but also activate various intracellular pathways that return to the mitochondria to induce apoptotic cell death, in part through regulation of pro- and antiapoptotic proteins such as the Bcl-2 family [151]. The Bcl-2 family is an essential group of proteins that regulate the integrity of the mitochondrial membrane, and is subdivided into three subgroups based on structural homology: antiapoptotic proteins including Bcl-2, Bcl-X_L_ and Bcl-w; proapoptotic proteins such as Bax and Bak and the BH3-only proteins including Bad, Bim, Noxa and PUMA [151]. An overweight of pro-apoptotic proteins at the membrane triggers the release of cytochrome c into the cytosol, which in turn combines with Apaf-1 and procaspase-9 to activate various caspases, such as caspase-3. The caspases are the proteins that perform the cellular degradation in apoptosis, exemplified by caspase-3’s cleavage of DNA repair enzymes leading to DNA damage [152]. Another feature of apoptotic cell death is the seemingly mandatory increase in expression of the so-called immediate early genes, such as c-Jun and c-Fos [153,154], which can be used as markers of apoptosis [155]. The importance of apoptosis in stroke is suggested by the neuroprotection afforded by increased expression of the antiapoptotic Bcl-2 [156,157] and by the ischemia-induced upregulation of proapoptotic proteins in animal models of cerebral ischemia [158].

Estrogens have been reported to reduce apoptosis in a number of studies. The antiapoptotic effects of estrogens include blocking the ischemia-induced reduction of Bcl-2 following MCAo [157,159], reducing caspase-3 after global ischemia [160], increasing expression of Bcl-2, Bcl-w and Bcl-X_L_ while decreasing Bax, Bad and Bim [161–167], attenuating injury-mediated DNA fragmentation [21], reducing the level of the 120 kDa caspase-mediated spectrin breakdown product [21], decreasing c-Fos induction [155], limiting apoptosis induced by staurosporine in cell cultures [168], inducing cGMP-dependent expression of thioredoxin—a redox protein with potent antioxidative and antiapoptotic properties [169]—and preventing glutamate-induced translocation of cytochrome c from mitochondria to cytosol (Figure 1) [170]. ER activation is also thought to limit apoptosis through increased expression of components in oxidative phosphorylation, making energy production more stable and thus maintaining mitochondrial membrane integrity [34]. Further, Bcl-2 over-expressing male mice sustained smaller infarct sizes compared to their male wild type counterparts, while this difference was not observed in females, which is likely to mean that apoptosis is one of the mechanisms of estrogen neuroprotection [157].

Considering the potential pathways for estrogens’ antiapoptotic actions, it is of interest that the Bcl-2 gene promotor has no ideal consensus sequence for an ERE, but that estrogens can interact with Sp1 for which there are several binding sites in the Bcl-2 gene promoter [171]. Also, estrogens have been shown to induce Bcl-2 expression through STAT3 and phosphoinositide-3-kinase/Akt-dependent CREB, which in turn possibly is activated by GPR-30 [164,168,172]. Akt also targets procaspase-9, members of the Forkhead family of transcription factors, which promote pro-death gene transcription [41]. These increases in antiapoptotic factors compared to proapoptotic factors afforded by estrogens are generally considered to convey neuroprotection by preventing activation of the permeability transition pore, thereby protecting against a cytosolic Ca^2+^-overload and release of cytochrome c into the cytosol [42,173].

Many of the aforementioned studies have been performed in animal models catering for *in vivo* relevant dose intervals in which the mechanism occurs. The reported antiapoptotic effects of estrogens presented above have been demonstrated using several different hormone administration protocols, though with an overwhelming dominance of low-dose and/or short-term regimens [15,17,21,155,159,165,166]. This corroborates the combined hypotheses that estrogens are neuroprotective through antiapoptotic mechanisms and that neuroprotection due to estrogens are mainly seen in low dose and/or short-term hormone regimens. However, to our knowledge no study has been performed where estrogens’ influence on apoptosis has been inhibited, and where the effect of such an inhibition has been assessed.

### 2.5. Growth Factor Regulation as a Mechanism of Estrogen Neuroprotection

Estrogens are known to regulate growth factors, an attribute that has been suggested as another mechanism for the hormones’ beneficial effects in cerebral ischemia [12,41]. Growth factors contribute to improved outcome after cerebral ischemia both by facilitating recovery and by decreasing apoptosis, thereby reducing infarct size [174]. This mechanism overlaps considerably with apoptosis, even if the extensive research focused on estrogens interaction with growth factors merits special attention. Also, the positive, possibly neuroprotective, effects of estrogens on neural cell proliferation, synaptogenesis, modulation of synaptic connectivity and regeneration [175,176] are probably mediated through regulation of growth factors and neurotrophins, including TGF-β, IGF-I, NGF, BDNF and NT-4 (Figure 1) [177–182].

17β-estradiol regulates the transcription of numerous growth factor genes through ERs’ binding to ERE in gene promoters. The factors influenced in this manner include, *i.e.*, VEGF [183], TGF-α [184], tau [185], BDNF, NT-4 and NGF [41,179]. ER not only co-localizes with and regulates the expression of neurotrophins and their cognate receptors, but estrogens and neurotrophins also share converging signaling pathways in the MAPK cascade, which includes activation of B-Raf and ERK, in turn regulating a broad array of cytoskeletal and growth-associated genes [186]. Additional evidence implying that estrogens exert their positive effects via growth factor interaction includes the cooperation with IGF-I to exert neuroprotection, possibly by sharing the MAPK and PI3/Akt signaling pathways [177,187]. Interestingly, IGF-I receptor blockade prevents estrogen neuroprotection while the ER antagonist ICI 182,780 can block IGF-I neuroprotection [188,189]. Similar results have been seen in models of cerebral ischemia [41,190], and in another study, a combination of IGF-I and 17β-estradiol did not add any extra protection against ischemia compared to the two substances administered separately [191], emphasizing to the relation between estrogens and growth factors as a protective mechanism in stroke. Moreover, estrogens have been postulated to promote recovery after stroke by directly regulating genes required for growth, such as tau microtubule-associated protein [185], GAP-43, [192], structural lipoproteins such as apolipoprotein E [193], and neurofilament proteins [194].

Thus, ample evidence exists for the notion that estrogens increase the activity of growth factors as a major mechanism for neuroprotection. As expected, the interactions of estrogens with growth factors have been demonstrated *in vivo* resulting from predominantly low-dose or short term estrogen administration regimens [15,17,178,180,181,187–189,192,194]. Thus there is good coherence between the biological environments in which the growth factor interactions have been shown and the notion that estrogens mainly are neuroprotective in physiological concentrations.

### 2.6. Vascular Modulation as a Mechanism of Estrogen Neuroprotection

The importance of vascular properties, such as vessel wall reactivity and contraction propensity, for the development of stroke is self-evident. Even though this category of factors may seem less important in animal models of cerebral ischemia where the vessel occlusion is artificial, it still influences the crucial aspects of collateral circulation and reperfusion. It is thus likely that increased vasodilatation in the cerebral vascular bed is beneficial in cerebral ischemia by facilitating blood flow to compromised brain regions [39]. The reactivity and contraction propensity of a blood vessel is strongly influenced by locally produced vasodilators including prostacyclin and NO, and vasoconstrictors such as endothelin-1, which in turn are regulated by other factors.

Estrogens have been shown to affect cerebral blood vessels in a number of studies; by relaxing cerebral arteries through inhibition of extracellular Ca^2+^ influx in vascular smooth muscle [195], moderating thrombotic mechanisms [196], influencing the biosynthesis of prostacyclin [197,198], potentiating ACh-induced endothelium-dependent relaxation [199], enhancing nNOS and eNOS levels [24,200–204] and thus increasing NO production [205–208], increasing COX-1 levels [200], and by less well characterized pathways which increase cerebral blood flow (Figure 1) [3,22,209–212]. It deserves mention that although eNOS could be neuroprotective through vasodilatation, it has also been shown to induce peroxynitrite formation under certain disease states [213], which in turn potentially could compromise cellular viability [214]. Most of these effects, such as influence on eNOS, COX-1 and prostacyclin synthase leading to vasodilatation and improved collateral flow, seem to be exerted via the classical genomic pathway or via the PI3/Akt pathway [39].

Several of the effects of estrogens on the cerebral vasculature have been demonstrated *in vivo* using estrogen regimens that are in the dose-range likely to mediate neuroprotection [15], such as low-dose subcutaneous injections [24] or physiologically cycling hormones [209]. There are therefore strong indications that estrogens affect important vessel properties in biological contexts relevant for the question of its effect on cerebral ischemia, even though evidence of this mechanism’s indispensability is lacking. Thus, to the best of our knowledge, no study has been performed where estrogens’ impact on blood vessels have been inhibited, and where the effect of such an inhibition has been assessed. Further, numerous studies have been unable to corroborate vascular effects of estrogens in stroke models, notably by absence of blood flow differences before or during MCAo between estrogen treated and estrogen deficient animals, even though differences in stroke outcome were observed [122,215–218].

## 3. Conclusions

### 3.1. Quality of Mechanism Experiments

Before an overall summary of the mechanisms dealt with here, brief considerations regarding study design, quality and causality may be in place.

In the process of elucidating which mechanisms are important for a certain biological target effect exerted by an investigated substance, different studies obviously contribute evidence of different weight, primarily depending on the experimental design. It is particularly difficult to draw conclusions about causality, exemplified by the fact that decreased/increased inflammatory response and oxidative stress resulting from estrogen supplementation may be a consequence of other mechanisms rather than a primary cause of the decreased/increased damage. Studies investigating mechanisms may be allocated three alternative ranks according to increasing degree of evidence:

1. The lowest degree of “evidence” for a certain mechanism comes from the discovery of a biological alteration, which *potentially* could bring about the biological target effect, in response to the investigated substance. An example is the finding that estrogens increase the concentration of the synaptic protein syntaxin, which *hypothetically* (without direct experimental evidence) could facilitate recovery after cerebral ischemia [219].
2. If the investigated substance has an effect on a presumed mechanism that in itself has been *proven* to exert the biological target effect the evidence is evidently stronger, even if the relative contribution of the mechanism cannot be quantified. An example is the fact that estrogens upregulate Bcl-2 [164], a protein in itself proven to decrease the damage from cerebral ischemia [156,157].
3. A yet higher degree of evidence for a mechanism’s importance is afforded when the presence of a specific blockage inactivates the biological target effect. An example is estrogens’ lack of protective effects in iNOS knocked out mice [111].

Two of the mechanisms of estrogens’ neuroprotection are supported by studies of the highest evidence rank, providing the best evidence for causality between the mechanism and the outcome. These are the abovementioned importance of iNOS [111,112] and the indispensable interactions between IGF-1 and estrogens [41,188–191]. Numerous studies in the current field do not reach the highest level of evidence, e.g., due to the lack of a suitable blocker, and thus the weight of the evidence in these studies needs to be adjusted accordingly.

### 3.2. Summary of Mechanism Evaluations

Decreased apoptosis, growth factor regulation, vascular modulation, *indirect* decrease of oxidative stress by altering the anti-oxidative defense and decreased inflammation have all been demonstrated in experimental settings fitting the dose-concentration range pattern established for neuroprotection and are thus likely candidates for being true protective mechanisms. Anti-inflammation (iNOS) [111,112], and interaction with growth factors (IGF-I) [41,188–191] are due to the abovementioned studies contributing with the highest evidence rank particularly well established. The *direct* anti-oxidative effect still needs to be demonstrated in relevant biological settings in estrogen concentration intervals known to be protective in whole-animal models, and this mechanism is, as aforementioned, especially difficult to assess because of the difficulties in separating genomic from non-genomic actions.

Of the suggested neurodamaging counterparts, only increased inflammation has been reported to occur under biological settings imitating conditions under which estrogens have been shown to be detrimental in whole-animal stroke experiments, and is thus to date the only real candidate of being a true damaging mechanism. The pro-oxidative effects, with their inherent problem of proving non-genomic actions, have as yet mainly been demonstrated in non-neuronal *in vitro* trials, and increased excitotoxicity has even less experimental support.

Thus while several mechanisms seem to contribute to the neuroprotective effects of estrogens in lower concentration ranges, it seems that possibly the anti-inflammatory effect of estrogens turning pro-inflammatory in supraphysiological concentrations could explain the observation that estrogens have opposing effects in different concentrations. It should however be emphasized that which mechanisms are true and false as assessed above is a highly complex issue, and that a review of this kind is better viewed as hypothesis-generating than hypothesis-testing.

### 3.3. Difficulties in Studying the Complex Estrogenic Mechanisms

When reviewing the abundance of studies investigating possible mechanisms of estrogens’ neuroprotective actions, risk of bias is evident in cases when different estrogen effects depending on its concentrations are not taken into account (relating to the concept of hormesis). Effects of estrogens assessed as potentially protective may sometimes just as well be interpreted as risks of increased damage. For example, in a study by Weiland, estrogens were found to increase NMDA binding sites in the hippocampus, which may be taken as a neurotropic effect (protective by facilitating recovery) or as a risk of increased excitotoxicity (harmful in cerebral ischemia) [127]. In another study estrogen replacement decreased the number of astrocytes and microglia in the hippocampus, which was taken as evidence for decreased inflammation instead of the possible alternative assessment that the estrogens induced neurodegeneration [110]. To minimize the risk of similar pitfalls, several controls are needed including e.g., the verification that the biological serum concentrations of estrogens are in the range proven to afford neuroprotection. Unfortunately, careful investigation of the administration methods used are scarce, and in the majority of instances when blood samples are drawn for analysis of serum estrogen levels the sampling is only performed at only one single time point (most often at animal sacrifice), which thus conveys little information of the serum concentrations before and after this specific moment.

### 3.4. Final Remarks

Investigations of different mechanisms for estrogens’ actions in stroke have been performed in a very wide range of concentrations, profoundly affecting the plausibility of the suggested mechanism given that the hormones’ neuroprotective and neurodamaging properties are in fact restricted to certain dose intervals. In future studies it is crucial that mechanisms are verified in relevant biological contexts where special care has been taken to control estrogen concentrations. This in combination with experimental designs catering for the highest level of evidence will provide the solid ground needed for characterizing estrogens’ actions and pathways in cerebral ischemia.

## Figures and Tables

**Table 1.** Administration method dose ranges in relation to neuroprotection and neurodamage. 17β-estradiol doses and capsule concentration ranges reported to induce neuroprotection and neurodamage, respectively, in rat models of cerebral ischemia. For injection regimens, daily doses are presented. Silastic capsules containing crystallized estrogen are omitted from the table, but were consistently neuroprotective [15].

**Administration method**	**Pellet dose/silastic capsule concentration/injection dose ranges**	
	Neuroprotection	Neurodamage
Slow-release pellets, subcutaneous	0.025–0.25 mg [19,20]	0.025–1.5 mg [6,8]
Silastic capsules filled with 17β-estradiol dissolved in oil, subcutaneous	180–4000 μg/mL [21,22]	Not reported
Injections, subcutaneous	10–5000 μg/kg BW [23,24]	Not reported
Injection, intravenous	10–1000 μg/kg BW [2,25]	Not reported
Injection, intraperitoneal	100–20,000 μg/kg BW [26,27]	Not reported
Injection, intramuscular	100 μg/kg BW [28]	Not reported
Infusion, intraventricular	50–150 μg [29,30]	Not reported
Oral administration	10 μg/kg BW [31]	Not reported

## List of Abbreviations

ACh: Acetylcholine
AMPA: α-Amino-3-hydroxyl-5-methyl-4-isoxazole-propionate
Apaf-1: Apoptotic Protein-Activating Factor-1
BDNF: Brain-Derived Neurotrophic Factor
BH3: Bcl Homology domain-3
CA1: Cornu Ammonis area-1
cAMP: Cyclic Adenosine Monophosphate
cGMP: Cyclic Guanosine Monophosphate
COX: Cyclooxygenase
CREB: cAMP Response Element Binding protein
eNOS: Extracellular Nitric Oxide Synthase
ER: Estrogen Receptor
ERE: Estrogen Response Elements
ERK: Extracellular signal-Regulated Kinases
GABA: Gamma-Aminobutyric Acid
GAP-43: Growth-Associated Protein-43
GPR-30: G-Protein coupled Receptor-30
GSK-3β: Glycogen synthase kinase 3β
IGF-I: Insulin-like Growth Factor-I
IL: Interleukin
iNOS: Inducible Nitric Oxide Synthase
IRA: Innovative Research of America
LPS: Lipopolysaccharide
LTP: Long Term Potentiation
MAPK: Mitogen-Activated Protein Kinase
MCAo: Middle Cerebral Artery Occlusion
MPO: Myeloperoxidase
NADPH: Reduced form of Nicotinamide Adenine Dinucleotide Phosphate
NFKB: Nuclear Factor Kappa-light-chain-enhancer of activated B cells
NGF: Nerve Growth Factor
NMDA: *N*-Methyl-d-Aspartate
nNOS: Neuronal Nitric Oxide Synthase
NO: Nitric Oxide
NT-4: Neurotrophin-4
PGE2: Prostaglandin E2
PI3: Phosphatidylinositol-3
PUMA: p53-Upregulated Modulator of Apoptosis
ROS: Reactive Oxygen Species
SOD: Superoxide Dismutase
Sp1: Specificity Protein-1
STAT3: Signal Transducer and Activator of Transcription 3
SVZ: Subventricular Zone
TGF-α: Transforming Growth Factor-α
TLR: Toll-Like Receptor
TNF-α: Tumor Necrosis Factor-α
VEGF: Vascular Endothelial Growth Factor
